# 

**Published:** 1888-08

**Authors:** 


					﻿American Medical Association.—The next meeting of the American
Medical Association will take place at Newport, Rhode Island, the first Tues-
day in June, 1887.
Receipted—For 1888—Dr. W. E. Searle, to July. For 1889—Drs. S. A.
McCuistion, to May; Tho«. J. Hendley, to January; J. E. Martin, to January;
W. Thornton, to January; L. Morgan, to January; E. Tate, to January; J.
Miller, to January; M. M. Talliaferro, to January; John M. Lemmon, to Jan.
Tulane Medical University.—The advertisement of this able school of
medicine can be found on page 15 of advertising department of this journal.
				

